# Assessing the Effects of Weather Conditions on Physical Activity Participation Using Objective Measures

**DOI:** 10.3390/ijerph6102639

**Published:** 2009-10-12

**Authors:** Catherine B. Chan, Daniel A. Ryan

**Affiliations:** 1 Departments of Physiology and Agricultural, Food and Nutritional Sciences, University of Alberta, Edmonton, AB, T6G 2R3, Canada; 2 Faculty of Science, University of the Fraser Valley, Abbotsford, BC, V2S 7M8, Canada; E-Mail: dan.ryan@ufv.ca

**Keywords:** physical activity, exercise, weather, seasons, winter, rain, snow

## Abstract

Habitual physical activity is an important determinant of health, yet many people are considered to be inactive. Identification of the obstacles to greater participation is necessary for the development of strategies to overcome those obstacles. The weather has been identified as a perceived barrier to participation in physical activity, but exactly which adverse weather conditions are most important, and the extent to which they contribute to decreases in physical activity have rarely been quantified in populations. In the past decade, a small number of studies have used publicly available databases to examine the quantitative effects of weather (e.g., temperature, precipitation, wind) on physical activity in children, adolescents and adults. This review examines our historical, qualitative versus emerging, quantitative understanding of how specific weather conditions affect a population’s activity.

## 1. Introduction

For those living in temperate climactic regions of the world, common sense suggests that changes in the ambient weather conditions may affect physical activity. High or low temperatures, rain, snow or wind may all serve to decrease the pleasure derived from outdoor activities. On the other hand, participation in some activities, such as skiing, skating or swimming outdoors may be enhanced by specific weather patterns. Gaining an understanding of the relationship between weather and health-related variables such as physical activity has increased in importance with the burgeoning prevalence of diseases for which physical inactivity is a risk factor, e.g., obesity, diabetes, cardiovascular disease and cancers. Furthermore, the effects of weather may interact with age, pre-existing disease conditions such as those named or others such as asthma, to exacerbate effects on physical activity. The weather cannot be changed, but knowledge of how weather conditions affect physical activity can help policy makers and providers of health care to adapt recommendations to mitigate its effects.

## 2. Impact of the Natural Environment on Health Indices

Elements of the physical environment are considered to be powerful determinants of health behaviors, thereby influencing population health [1] and have been categorized as “barriers”, “facilitating conditions” or “contextual influences” [2]. In the context of health, the potential of both the natural [3] and built environments [4] to influence behavior is increasingly acknowledged and taken into account by urban planners and others [5]. The natural environment encompasses factors such as the terrain, vegetation and weather [6]. Quantifying the impact of these variables has recently been facilitated by the development of affordable and accessible technologies such as geographic information systems and access to databases through the internet.

Association between non-infectious health conditions and the weather has been identified in a general sense, using the seasons as overall indicators of weather patterns. For example, worsening of glycemic control in diabetic patients correlates with the winter season [7], as does a reduction in high-density lipoprotein (HDL)-cholesterol [8,9]. In a study of nearly 150,000 Austrians, the winter season was associated with hypertension, total cholesterol and body mass index, increasing the risk of chronic coronary disease by 6.8% in men and 3.6% in women [10]. The authors postulated a physiological effect not caused by changes in living conditions because of the ubiquity of the problem across the population. Physiological effects could be stimulated by seasonally-influenced parameters such as day-length, which influences hormone secretions. Conversely, the factors such as changes in dietary patterns or work- or leisure-time physical activity, which may be influenced by seasonal weather changes, could also affect health. Seasonal variations in diet have been identified in Chinese women [11] and Spanish men and women [12] but the studies were not designed to correlate with health indices. Seasonal variations in lycopene and beta-carotene intakes were not related to glucose control in type 1 diabetic children [13] but overall the relationship between seasonal dietary variation and health has not been well-documented. One exception is the seasonal variation in vitamin D concentrations in serum, which is a function of both intake of fortified foods and sunlight exposure. Vitamin D status is associated with increased incidence of several cancers, type 1 diabetes, osteoporosis, among other diseases [14]. The question of how best to assess the effects of weather versus other seasonally-influenced variables is of interest to those trying to incorporate the realities of the physical environment into urban planning, development of health interventions, disease prevention strategies, and the like.

## 3. Qualitative Assessment of the Effects of Weather on Physical Activity

Historically, many of the first studies of the potential effects of weather or season on physical activity were included in assessments of self-reported perceptions of the effects of physical environment parameters. Thus, in 2002 Humpel *et al.* [1] identified 19 studies published between 1989 and 2001. Of those, three studies included “poor weather” or “lack of good weather” as one of the environmental variables queried, but none identified weather as a determinant of physical activity [1]. Subsequently, the weather was explicitly examined as a factor in walking for different reasons using a mailed survey. Those who were least likely to perceive the weather as a barrier were more likely to be high-volume walkers, whether for exercise or general walking about their neighborhoods [15]. A sampling of other studies published since that time solidify the conclusion that people from a variety of populations perceive inclement weather to be a barrier to physical activity. Neither socioeconomic status nor ethnicity altered the perception of the weather as a deterrent to recreational physical activity in Australian adults, although the sample size in both studies was small (<75 people) [16,17]. People with chronic disease, such as asthma, perceive weather to influence their ability to exercise [18] and it was identified as a factor in low compliance in physical activity interventions [19]. Low-income mothers in the United States regarded weather as an impediment to physical activity [20,21], as did Australian mothers [22]. The perceptions of parents influence the activity of their preschool-age children [23] but even 6–8-year-old children depict good weather being compatible with being physically active [24]. These qualitative studies clearly identify the weather as one element of the physical environment perceived to have an impact on physical activity but do not address the specific types of weather that are problematic, nor the magnitude of effect exerted by various weather conditions.

## 4. Identification of Applicable Literature

To find the publications that studied the effects of either seasons or weather on physical activity we conducted a search of PubMed using the search terms “physical activity”, “exercise”, “seasons”, “month”, “weather”, “rain”, “snow”, “precipitation”, and “winter”. Review articles captured in this scan were also reviewed for relevant articles, leading to a total of 72 initially identified articles. With respect to studies of the effects of seasons, the studies ultimately selected were not exhaustive because this was not the main focus of this paper; moreover, a recent review [25] provides an excellent summary of these studies up to 2006. For our purposes, objective measures of activity related energy expenditure by doubly-labelled water technique, or objective measures of physical activity by accelerometer or pedometer were preferred, resulting in inclusion of 17 papers. For the studies of weather, objective measures of climatological conditions was required for inclusion for a total of 10 reports, 8 of which also used objective methodology to ascertain physical activity. A summary of the 27 publications is given in Table 1.

## 5. Quantitative Assessment of the Effects of Season on Physical Activity

Using the four seasons as a surrogate provides clues as to which specific weather elements affect physical activity, measured semi-quantitatively by means of questionnaires or quantitatively with accelerometers or pedometers. From this body of work, the winter season in temperate climates is correlated with reduced physical activity. An example of one of the larger studies is the Framingham Offspring Cycle conducted in the northeastern United States, which analyzed physical activity by questionnaire, compared with seasons from 1979–1983 [49]. The data show that both men and women are more physically active during leisure time in the summer than in the winter. Moreover, it provides an analysis of the types of activities adopted by season. A 2–3-fold greater volume of walking for pleasure, the most prevalent type of activity for both men and women, was reported in spring-summer-fall seasons, compared with winter. In addition, outdoor activities such as gardening and lawn-mowing tended to replace indoor pastimes such as home exercise and bowling [49]. Data from the Canadian Community Health Survey, Cycle 2.2 of more than 20,000 Canadians collected in 2004 revealed that the number of inactive respondents increased from 49% in summer to 64% in winter [50]. Smaller studies, as well as those from other countries, and those concentrating on adolescents or children, tend to replicate these findings [25], with some exceptions. In areas of the United States with hot, humid summer weather, physical activity of children was in fact lower in summer than winter [51]; a small study in the United Arab Emirates also suggested that extremely hot weather would decrease physical activity although a seasonal analysis was not conducted [52].

In some reports, the magnitude of seasonal variation in physical activity was estimated from the survey data collected. As noted above, the volume of walking for pleasure contracted by 50–65% in winter in the northeastern United States. When expressed as kcal/week, activity related energy expenditure was 40% lower in winter [49]. This is similar to the 31% winter deficit in activity related energy expenditure calculated from Canadian data [50] and the 35% decrease noted. Participants in the Seasonal Variation of Blood Cholesterol Study, 1994–1998 accrued 1.4 (men) or 1.0 (women) metabolic equivalent (MET)-hours/day more in summer than winter, as estimated from questionnaires, although there was virtually no seasonal fluctuation in those not engaging in some level of recreational physical activity [26]. In this study, physical activity was also objectively measured using accelerometers, which demonstrated decreases in moderate or greater intensity physical activity (>20 counts/min on the accelerometer) in winter of 51 min/day in women and 16 min/day in men [26].

In general, the use of methodologies that objectively quantify physical activity variation by season complement and confirm the results primarily obtained using questionnaires. The energy expenditure of high school students in France was measured using whole-body calorimetry. Twenty-one percent of the variation in daily energy expenditure was explained by the season, with autumn being highest [27]. An ethnically diverse sampling of children in the United States experienced a 0.42 MJ/day decrease in total energy expenditure in fall compared with spring as measured by the double-labelled water technique; this was almost entirely accounted for by changes in activity-related energy expenditure [28]. A sample of nine men in the United Kingdom had non-significant seasonal variation in activity measured by calorimetry of 1.88 x basal metabolic rate in winter versus 1.96 x in summer [29]. Likewise, a Dutch cohort of men and women exhibited no difference in total energy expenditure measured by doubly-labeled water but a seasonal decrease in physical activity level (PAL, i.e., total energy expenditure divided by resting metabolic rate) and activity-related energy expenditure in winter. The winter decrement in PAL was more pronounced in men than women and was detectable only in those who were most active in the summer; those who were inactive in summer remained inactive in the winter [30].

Despite being the gold standard, using doubly-labeled water to measure energy expenditure is expensive and thus limits sample size. Accelerometers and particularly pedometers are more affordable and therefore permit larger sample sizes, with accelerometers measuring a wider variety of activities as well as intensity whereas pedometers are limited to measurement of walking. In the last five years, we identified 12 studies that correlated accelerometer or pedometer-measured physical activity with the seasons.

In adults in the Netherlands, the United Kingdom and the United States, seasonal variation in walking were identified. The Dutch study reiterates an important caveat of equating “season” with “weather”, because in that study the amount of physical activity was proportional to the number of hours of daylight [31]. A winter steps/day difference of approximately −1,300 compared with summer was quantified in 96 adults studied in the United Kingdom, equating to a 13% deficit [32]. In comparison, 23 American adults living in two states with relatively mild winters had an approximately 10% lower step count in December than September [33]. These latter two locations varied in both weather extremes and latitude (day length changes), yet the degree of change was similar. Of interest is a recent study showing that enrolling older women in a physical activity intervention negated the seasonal variation in steps/day previously observed [34]. Given that developing interventions that overcome the winter physical activity deficit is a goal of several research groups working in the field, these results are encouraging.

Similar to adults, surveys of adolescents and children using accelerometers or pedometers bolster the evidence that seasons affect physical activity. These reports come from Cyprus [35], Scotland [36], the United States [37,38], Australia [39] and the United Kingdom [40]. In most cases, reduced physical activity was quantified in winter. However, studies of children are confounded by the amount of time they spend in school in fall-winter-spring versus the summer. Thus, Lanningham-Foster *et al.* identified a significant difference in physical activity of children on summer vacation compared with those in a traditional school setting. This difference disappeared when children went to a school with an “activity-permissive” environment [41].

## 6. Quantitative Assessment of the Effects of Daily Weather Variations on Physical Activity

The studies documented above lead to the conclusion that the perceived negative effects of bad weather on physical activity are true, assuming that the strong evidence for a winter-related decrease are caused by weather-related variables and not by day-length. However, winter-related decreases in physical activity are noted across a variety of climactic zones, which suggests that other factors may also play a role. A relatively smaller body of literature now addresses the question of specifically what weather elements may account for reduced physical activity throughout the year. Most of these are combined with quantitative measures of physical activity.

Matthews *et al.* [26] reported the associations of specific weather activity with physical activity in the Seasonal Variation of Blood Cholesterol Study conducted in Massachusetts, United States. Meteorological data was obtained from the National Oceanographic and Atmospheric Administration’s (NOAA) National Climatic Data Center at the airport nearest the study location, and included average daily temperature, barometric pressure, humidity, the type and amount of precipitation and average daylight cloud cover. These data were correlated with self-report of household and leisure-time physical activity on a monthly basis. Periods of peak household and leisure-time physical activity overlapped with periods of least cloud cover and snow (but greater rain), and warmest temperatures. However, within the overall pattern, smaller but distinct sub-patterns were detected. For example, days of solid precipitation in the winter months coincided with greater physical activity among men [26]. While this study established the potential for specific weather patterns to affect physical activity, the effects of day-to-day variations were not addressed.

A cross-sectional analysis of randomly selected, phone-interviewed Americans living in North Carolina and Mississippi was geo-coded by address and matched with ambient weather conditions over a 30 day period concurrent with the recall survey of physical activity. Climatological data were obtained from the National Climactic Weather Center of NOAA in each study area. Average windchill, heat index, standard pressure and total precipitation (mainly rain) were obtained and a “weather score” created to reflect weather severity, which was averaged over 30 days and then divided into quartiles for analysis. No correlation was detected between objectively measured weather and self-reported physical activity [6]. Given that the general approach in [26] and [6] was similar, the differences in outcome may be related to smaller differences in day-to-day weather in North Carolina and Mississippi than Massachusetts.

Attendance of older women at an exercise class was used as an objective measure of the effects of weather variables on physical activity [42]. Weather information was obtained from the Purdue University Applied Meteorological Group, including hourly information of precipitation type and intensity, as well as the degree of windiness. Indices were created to reflect weather severity with respect to wind-chill, heat, degree of overcast, rain or snowfall. Class attendance was compared with the degree to which each index varied from average in the five hour period preceding the class. Humidity was included in the model as a continuous variable. This novel approach found that temperatures above 90 °F a wind-chill index below 20 or any amount of snowfall contributed to a 25–40% lower attendance. In contrast, a sunny day or a day with higher atmospheric pressure was associated with a 10–25% increase in attendance [42].

High-school students from Montreal, Canada reported physical activity in a 7-day recall, which was then compared with both season and specific weather elements. Weather conditions for the local airport, which was within 50 km of all schools in the project were obtained from the Environment Canada website. Seasonally, physical activity in April–June was nearly 20% higher than the reference month, December, which was similar to November–March. Effects of weather on physical activity in December were reported as a 6% increase for a 10 °C increase in temperature, an 11% decrease for 10 cm of snowfall and a 3% decrease for 10 cm of snow on ground. Rainfall had no effect (but was probably rare in December, which had an average temperature of −12 to −15 °C) [43]. Students in this sample were followed for five years, during which time overall physical activity declined significantly. The authors suggest that the winter physical activity deficit is not fully recovered in the following summer-spring, contributing to the well-documented decline in physical activity through adolescence [43].

Long-term, continuous, objective monitoring of both physical activity and weather has been undertaken by three groups. The Nakanojo Study [44] followed 41 healthy, elderly Japanese men and women (mean age 71 years) for 450 days. Study participants wore electronic physical activity monitors with 36 day memory 24-h each day. Steps data were retrieved by study personnel each month. Meteorological data were obtained from the local meteorological station and included daily mean temperature and wind-speed, total precipitation (mainly rain) and duration of bright sunshine. The geographic location appeared to be subject to considerable rainfall, with accumulation of 25 mm or more on 29 days during the recording period (as estimated from the data presented). The effect of rainfall on steps/day was exponential and negative. The remaining weather elements were therefore only analyzed on days when the rainfall was <1 mm, revealing a quadratic relationship between mean temperature and steps/day, with physical activity increasing up to 17 °C and then declining again [44]. The effects of day-length and humidity were very modest and not considered to be of any practical significance. Wind effect was not significant, but wind-speeds observed were very low (generally less than 10 kph).

The physical activity of 177 younger adults (mean age 44 years) was monitored in a Canadian study that was part of a pedometer based intervention to increase walking. Individual contributions of steps/day to the weather study database averaged 64 days [45]. Meteorological data for the local area were obtained from Environment Canada’s website and included daily total rainfall and snowfall, mean temperature, maximum wind-speed, and accumulated snow on ground. The relationship between steps/day and weather elements was defined using a mixed linear model and controlled for body mass index (BMI), gender, and the number of days each individual had been in the intervention. It also controlled for weekday, month and the interaction between weekday and month. The geographic location (Charlottetown, Prince Edward Island) experienced less rainfall than the Japanese location, but had temperatures averaging below 0 °C between December and March and 20–60 cm of snow each month between December and April in the years studied. Small amounts of rain (5 mm) decreased steps/day by 5.2%, progressing to a decrease of 8.3% with 14 mm of rain, with no further increase thereafter. Increasing temperature was associated with increasing steps/day, with a 2.9% increase for each 10 °C increase in temperature. Effects of rain and temperature were independent of BMI and gender, as was accumulated snow on ground, which decreased steps/day by 3.6% for every 10 cm accumulated. Daily snowfall had different effects depending on both BMI and gender, such that males with a BMI of 20 kg/m^2^ had a 21% increase in steps/day after 10 cm snowfall but heavier males had no significant change in physical activity. In contrast, females with a BMI of 20 kg/m^2^ had no change in steps/day and heavier women experienced a significant decrease of approximately 10%. These data appear to validate less quantitative reports showing men from the Northeastern United States reporting peak vigorous household activity in January [53], when snow removal and wood-chopping were also reported to occur among men [49]. Windy days, estimated by the maximum recorded wind-speed in 24 h, decreased steps/day the most in leaner individuals and least for those with a BMI >30 kg/m^2^, ranging from 5% to 2% with a 20 km/h wind. From these data, the authors estimated that inclement weather has the potential to reduce physical activity by up to 20%, on a day that was both cold and rainy, for example.

Rainfall and mean temperature also had significant impacts on the physical activity of children [46]. School children (n = 1,251) in Auckland, New Zealand wore pedometers for three weekdays and two weekend days. Meteorological data were obtained from the weather station closest to the child’s school and were provided by the National Meteorological Service. Total rainfall, mean daily temperature, mean wind-speed and duration of bright sunshine were recorded and relationships with physical activity were deduced using mixed modeling, with adjustments for weekday-weekend, grade level and socioeconomic status. Data for boys and girls were analyzed separately. For boys, weekend days were more affected by a 10 °C decrease in temperature, with a decrease in steps/day of >3,000, and the greatest effect was in the lowest socioeconomic group. Rainfall effects were smaller and similar across days of the week and socioeconomic groups, estimated at −1,700 steps on a day with 5 mm rain. In contrast, girls experienced a decrease of 2,300 steps/day for a 10 °C decrease in temperature on weekdays but no change on weekend days. Effects of rainfall were similar to that of boys. Other weather elements had effects that were considered trivial or unclear.

A comparison of the three quantitative studies discussed is given in Table 2. Given the diverse populations studied (adults, elderly adults, children) under differing conditions (in an intervention, retired, in school) it is not surprising that differences in the magnitude of effects of weather elements on steps/day were observed. More data need to be gathered to determine if, in fact, the elderly are more susceptible to mild fluctuations in weather or whether the smaller magnitude of changes in the adult group are because the participants were in an intervention to increase physical activity and therefore had higher motivation to exercise in spite of weather conditions. Furthermore, all of these studies are likely to underestimate the degree to which weather affects physical activity because the weather was averaged over 24 h and the activity accumulated during waking hours. Thus, if it was rainy and windy during the night, but cleared up during the day, physical activity was probably little affected.

The issue of real-time effects of weather on physical activity was addressed by direct observation of people in Ohio, United States compared with meteorological conditions at the time [47]. Periods of precipitation were not included in the observations. Weather data were obtained from an Automated Surface Observing System at the local airport operated by three federal agencies. Data on precipitation, wind-speed, relative humidity, atmospheric pressure, temperature and dew point were obtained. Apparent temperature was calculated to take into account effects of humidity and wind on heat balance. Using univariate analysis, on weekdays, temperature and apparent temperature increases correlated positively with the number of walkers on a track whereas relative humidity and barometric pressure was negatively correlated. The latter two variables also decreased the time spent walking on the track. However, joggers on the track were not influenced by any type of weather, similar to either walkers or joggers observed on sidewalks or streets. The number of children walking to school and the number of bicyclists was decreased only by wind-speed. On the weekend, weather variables did not significantly affect the number of people walking, jogging, bicycling or dog-walking. Multiple linear regression models showed that 58% of the variance in the number of walkers on the track was accounted for by apparent temperature, dew point and barometric pressure. A 4 °C decrease in temperature was equated with a 40% reduction in the number of walkers on the track [47]. While this study is the first to illuminate direct effects of weather on activity in real-time using objective measures, it does not reveal whether a reduction in physical activity at a particular point in time is made up for later. The data suggest that the purpose for which the activity is being undertaken (exercise versus active transportation, for example) influences the impact of the weather.

Less personal interrogation of how weather affects physical activity has also been reported. Lindsey *et al.* [48] monitored usage of urban trails in Indiana, United States compared with weather characteristics. Trail traffic was detected using infrared monitors. Weather data were obtained from NOAA, compared with long-term daily averages, and expressed as deviations from average of temperature, precipitation, snow and hours of sunshine. Trail use peaked in the summer months. All of the weather elements had significant effects in the expected directions on trail use. An increase in mean daily temperature of 1 °F (0.6 °C) above average increased trail traffic by 3.2% while an inch (25 mm) of precipitation above average decreased usage by 40%. While the use of infra-red monitoring is of interest for gathering information on populations, the failure to correlate weather with activity in real time is a limitation.

## 7. Conclusions

In the past decade, the ability to objectively measure both physical activity and correlate it with weather events has furthered our understanding of how the natural environmental can have an impact on activity and, potentially, on human health. Different strategies have been used to monitor the physical activity of individuals (pedometers, accelerometers) versus populations (infrared monitors, direct observation). Weather information is now readily obtainable from data repositories available from national weather services, which is often easily accessed on the internet, and can be correlated with physical activity in real time. To date, the number of published studies is small but in general the data confirm the perception that precipitation has the largest correlation with physical activity. This correlation is generally negative but snow may, in fact, increase physical activity in men. In addition to gender, body mass index, socioeconomic status, the purpose of the activity and the age of those observed have been identified as potential contributing factors. Epidemiologists should control for season and weather because they significantly affect physical activity in a variety of populations. Furthermore, all of the reports to date have been observational studies; thus, causation is inferred but not proven.

Additional studies, conducted across a range of climactic zones, will be helpful in developing physical activity promotional materials and interventions that take the weather into account. Those developing physical activity interventions that utilize outdoor spaces and facilities need to consider how to counter-act the negative impact of precipitation; for example, by suggesting alternative indoor activities and emphasizing the need for protective clothing and proper footwear. Alternatives to walking, such as skating, snow-shoeing and cross-country skiing, can be promoted in cold climates to take advantage of the snow. Furthermore, the limited data suggesting that individuals in an intervention may be motivated to continue despite inclement weather merits further investigation as to how this potential can be maximized. An interesting experiment would be to combine the approach of Humpel *et al*. [15] examining the “physical activity personality” of participants (e.g., “neighbourhood walkers”, “high-volume” walkers) versus objectively measured, real-time physical activity using an accelerometer compared with real-time weather data. This might help reveal traits that could be built upon to develop physical activity interventions that work. Lastly, the effects of weather on disease severity have yet to be objectively quantified. Dasgupta *et al*. [54] have described the design of a study to follow type 2 diabetes patients through four seasons, measuring weather variables and walking by pedometer to correlate with hemoglobin A1C (an indicator of glycemic control) and blood pressure.

## Figures and Tables

**Table 1.** Effects of the seasons or objectively measured weather on physical activity.

**Location and population**	**Weather- or season-related variables**	**Method of physical activity measurement**	**Year of data collection**	Citation*
Massachusetts, USA; adults, n = 580	Weather: Mean daily temperature, barometric pressure, relative humidity, total daily precipitation, average cloud cover during daylight hours	Accelerometer (total physical activity) and 24-h recall questionnaire (leisure-time physical activity)	1994–1998	Matthews *et al.*, 2001 [26]
France; children, n = 83	Seasons: spring versus autumn	Whole body indirect calorimetry (total activity in 24 hours)	Not stated	Bitar *et al.*, 1999 [27]
Vermont and Alabama, USA and Guatemala City, Guatemala; children, n = 232	Seasons: spring, summer, autumn	Doubly labeled water (total, resting and activity-related energy expenditure)	Not stated	Goren *et al.*, 1998 [28]
Scotland, U.K.; men, n=10	Seasons: summer versus winter	Indirect calorimetry (activity-related energy expenditure)	Not stated	Haggarty *et al.*, [29]
Maastricht, Netherlands; adults, n = 25	Seasons: summer versus winter	Doubly labeled water (total energy expenditure) and respiration chamber (sleeping metabolic rate) – deduced physical activity level and activity related energy expenditure	Not stated	Plasqui and Westerterp, 2004 [30]
Netherlands; adults, n = 134	Seasons	Accelerometer (total activity during waking hours)	Not stated	den Hoed *et al.*, 2008 [31]
United Kingdom; adults, n = 96	Seasons: summer versus winter	Pedometer (total activity)	2005–2006	Hamilton *et al.*, 2008 [32]
South Carolina and Tennessee, USA; adults, n = 23	Seasons: summer, autumn, winter, spring	Pedometer (total activity during waking hours)	Not stated	Tudor-Locke *et al.*, 2004 [33]
Pennsylvania, USA; women, n = 508	Seasons: summer, autumn, winter, spring	Pedometer (total activity during waking hours)	2002–2004	Newman *et al.*, 2009 [34]
Cyprus; children, n = 256	Seasons: summer versus winter	Pedometer (total activity)	Not stated	Loucaides *et al.*, 2004 [35]
Scotland, United Kingdom; children, n = 209	Seasons: summer, autumn, winter, spring	Accelerometer (total activity during waking hours subdivided by intensity categories)	2001–2002	Fisher *et al.*, 2005 [36]
Southern USA; children, n = 401	Seasons: winter versus spring	Pedometer (total activity during waking hours)	2005	Beighle *et al.*, 2008 [37]
Massachusetts, USA; children, n = 35	Seasons: autumn, winter, spring	Pedometer (total activity)	2004–2005	Vadiveloo *et al.*, 2009 [38]
Melbourne, Australia; children, n = 540	Seasons: spring versus summer	Accelerometer (total activity subdivided by intensity categories)	2001 and 2004	Cleland *et al.*, 2008 [39]
England, U.K.; children, n = 5595	Seasons: summer, autumn, winter, spring	Accelerometer (total activity subdivided by intensity categories)	2003–2005	Riddoch *et al.*, 2007 [40]
Minnesota, USA; children, n = 24	Seasons: summer versus school year	Accelerometer (school day activity or total activity during waking hours)	Not stated	Lanningham-Foster *et al.*, 2009 [41]
North Carolina and Mississippi, USA; adults, n = 1482	Weather: hourly observations of temperature, dew point, atmospheric pressure, wind speed, precipitation; calculated wind chill, heat index in daytime hours. Data used to create a daily “weather score”.	Self-reported leisure-time physical activity	2002–2003	McGinn *et al.*, 2007 [6]
Indiana, USA; women, n = 110	Weather: hourly assessment of presence and intensity of sunshine, rain, snow, fog, hail, freezing rain, blowing snow, temperature, atmospheric pressure on the day of the exercise class. Data used to create heat index and wind chill.	Attendance at an exercise class	Not stated	Tu *et al.*, 2004 [42]
Quebec, Canada; children, n = 1293	Seasons: summer, autumn, winter, spring Weather: daily mean temperature, total rainfall, total snowfall, ground accumulation of snow	7-day recall survey of leisure time physical activity	1999–2004	Bélanger *et al.*, 2009 [43]
Nakanojo, Japan; adults, n = 41	Weather: daily mean temperature and wind speed, total sunshine, total precipitation, relative humidity	Pedometer (total activity)	2001–2002	Togo *et al.*, 2005 [44]
Prince Edward Island, Canada; adults, n = 177	Weather: daily mean temperature, daily maximum wind speed, total rainfall, total snowfall, ground accumulation of snow	Pedometer (total activity during waking hours)	2002–2003	Chan *et al.*, 2006 [45]
Auckland, New Zealand; children, n = 1115	Weather: daily mean temperature, total rainfall, mean wind speed, hours of bright sunshine	Pedometer (total activity during waking hours)	2004	Duncan *et al.*, 2008 [46]
Ohio, USA; children and adults	Weather: hourly precipitation, wind speed, relative humidity, atmospheric pressure, temperature, dew point	Direct observation at specific locations (residential streets/sidewalks, school transportation routes, outdoor oval tracks)	Not stated	Suminski *et al.*, 2008 [47]
Indiana, USA; children and adults	Weather: expressed as deviations from average temperature, precipitation, snowfall, sunshine	Infra-red monitors on greenway trails	2001–2005	Lindsey *et al.*, 2006 [48]

* In order of appearance in Sections 5 and 6.

**Table 2.** Summary of effects of weather elements on steps/day.

	**% Change from baseline**			
	**5 mm rain**	**10 cm snow**	**+10 °C**	**+20**–**25 kph wind**
Togo *et al.* [44]	–26	n/a	+13 (from 1–11°C)	n/a
Chan *et al.* [45]	–5.2	+21.1 (lean men) −9.6% (obese women) p > 0.05 (obese men, lean women)	+2.9	–5.2 (lean men and women) –2.8 (obese men and women)
Duncan *et al.* [46]	–11 (boys, weekday) –8.3 (girls, weekday)	n/a	+11 (boys, weekday) +16 (girls, weekday)	ns

n/a: not applicable; ns: not significant.
